# Inequality, Trust and Fear: Migrant Healthcare During the COVID‐19 Pandemic and Beyond

**DOI:** 10.1111/1467-9566.70077

**Published:** 2025-08-02

**Authors:** Alexis Hawthorne, Alicja Bobek, Lina Sandström

**Affiliations:** ^1^ Centre for Diversity, Policy, Research and Practice Oxford Brookes University Oxford UK; ^2^ School of Global Studies University of Sussex Brighton UK; ^3^ AIB Research Centre on Inclusive and Equitable Cultures (RINCE) Technological University Dublin Dublin Ireland; ^4^ School of Humanities Örebro University Örebro Sweden

**Keywords:** COVID‐19, discrimination, Europe, health, inequality, migrant, pandemic, trust

## Abstract

Many migrants are considered to be disadvantaged regarding their social, economic and health outcomes. During the COVID‐19 pandemic these inequalities grew starker, especially in healthcare, as migrants were at increased risk of exposure and had a reduced ability to seek care or access support. This paper will explore these issues through the analysis of narrative interviews gathered during a large‐scale, cross‐European project which explored the experiences of 740 marginalised individuals, including migrants, during the COVID‐19 pandemic. We build upon Beck's concept of the ‘risk society’ by exploring the ways in which neoliberal states have created exposure to risk; however, we also adopt a critical approach in examining how risk is not distributed equally. The following themes were revealed: first, migrants were often more exposed to the virus due to their occupational status. Second, migration status had an impact on access to healthcare, with undocumented migrants especially vulnerable. Third, the intersection between health crises and trust was identified: on the one hand, migrants lack of trust in host country institutions affected their engagement with services; on the other, they also experienced a lack of trust in them, as they were often perceived as a ‘risk’ in relation to the virus.

## 1 Introduction

The COVID‐19 pandemic has notably widened gaps in service provision for the most vulnerable in society as it exacerbated existing inequalities and created new forms of inequity, with migrants considered as one of the most vulnerable groups (Bobek and Sandström 2024; Fasani and Mazza 2020). In addition to facing inequalities related to their labour market position, migrants also experienced unequal provision of health services and state support (Reid et al. 2021). Although the crisis heightened anxieties and concerns regarding health for many, migrants felt this more acutely due to the additional challenges they faced (Crouzet et al. 2022). These were related to the working conditions characterised by higher exposure to the virus (Miles et al. 2024), pressures to continue work which was particularly acute for low‐paid and undocumented migrants (Miles et al. 2024) and limited access to services for those with unregulated status (Mengesha et al. 2022). More broadly, information regarding the pandemic related to health, work and movement restrictions was often lacking for migrant groups due to language barriers, the digital divide and/or a pre‐existing lack of understanding regarding bureaucratic and health system processes in their host countries (Zlotnick et al. 2022; Jiménez‐Lasserrotte et al. 2023). Migrants also felt wary of possible discrimination from service providers, and suspected vaccine and tracking technology to be a means of potential deportation (Younis 2021; Orton et al. 2022).

In this article we discuss these issues, with particular focus on mutual trust, access to health services and exposure to the virus. Further, we reflect upon the ways in which entrenched societal precarity was focused upon the ‘socially weakest groups’, who were expected to perform the most dangerous tasks within society, even prior to the COVID‐19 pandemic (Woolfson and Likic‐Brboric 2008). Our argument builds upon Beck's (1992) concept of the ‘risk society’ by exploring the ways in which neoliberal states have created exposure to risk, whereas the pandemic constituted a new form of ‘world risk’ (Beck 2007). However, we also adopt a critical approach to Beck's thesis that the risk society replaces class structures by demonstrating how risk, and the ability to shield oneself against risk, is not distributed equally and those with the least power in society, such as migrants, are perceived as creators of risk due to societal anxiety and fear (Akay and Palabıyık 2021), leading to mutual distrust between citizens and migrants during crisis. Although Beck (1992) argues that knowledge has now replaced wealth as a powerful commodity allowing individuals to mediate risk, the pandemic demonstrated that wealth was in fact still vital as it allowed those who were more financially secure to either have employment that allowed them to transition to working from home, or to withstand a loss of continuous income, thus shielding them from the virus itself, or from financial disruption. As is evident within the narratives in this article, migrants were often unable to access these ‘shields’, and our paper thus contributes to existing literature exploring how the pandemic was a form of globalised ‘anthropological shock’ through which the complexities of risk and distrust were brought to light (Chan 2023).

The analysis presented in this paper is based on the findings from the pan‐European RESISTIRÉ Project (2021–2023), the main objective of which was to examine the impact of COVID‐19 policies on gender+ inequalities in Europe. Migrants were one of the groups considered in the study, and access to healthcare was among the core topics explored by the project. The research questions that prompted the following analysis were how are vulnerable groups affected by the pandemic? How do they respond to inequalities created or exacerbated by the crisis? The results of the analysis are contextualised through the context of mutual trust and the stigmatisation of migrants during the pandemic. We thus contribute to existing literature on the relationship between migration status and healthcare not only during the pandemic, but in general, with crisis utilised as a context to expose barriers related to migrants' ability to access services, and diminished mutual trust. In this context, we link trust with the concept of risk as migrants, during crises, may represent the ‘dangerous’ other. This lack of trust is also exacerbated by different understandings of reliable knowledge that, during the pandemic, was constructed on a national level regarding scientific explanations of the virus and was disseminated to the populus in unequal ways. This work provides insight into the ways in which qualitative methodologies can shed light upon the deepening of pre‐existing, and the creation of new, inequalities during the COVID‐19 crisis for migrants.

The paper is structured as follows. We first provide an overview of existing literature on migration, healthcare and trust; followed by the RESISTIRÉ methodology. We will then present our analysis of the qualitative narrative interviews collected as part of the project. Final sections provide discussion and conclusion.

## 2 Literature Review

### 2.1 Migration Status and Exposure to the Virus: Living and Working Conditions

The existing literature on migrants and COVID‐19 exposes multiple issues related to migration status and healthcare. First, it has been demonstrated that some migrants were at an increased risk of exposure to COVID‐19 infection due to their housing conditions which prevented them from social distancing (Jiménez‐Lasserrotte et al. 2023). Second, higher risk of contagion was also evident in the work environment, especially for those who were concentrated in high‐risk, face‐to‐face occupations (Côté et al. 2021), whereas studies demonstrated that migrants were also more likely to be in jobs which were hard to transfer to teleworking (Fasani and Mazza 2020; Côté et al. 2021). Interestingly, the higher prevalence of the so‐called 3D jobs (dirty, dangerous and demeaning) among migrants also ‘shielded’ native workers from negative economic shocks and health risks (Bossavie et al. 2022). Thirdly, as a proportion of these were also seasonal workers, undocumented or with temporary visas, their status and access to support was also tied to their employer (Reid et al. 2021). Such workers were often not able to demand safety procedures, and were ineligible for sick leave or COVID‐19 support payments (Reid et al. 2021). All these factors should be considered in the context of limited access to healthcare services, which will be discussed in the following sections.

### 2.2 Migrants and Healthcare During the COVID‐19 Pandemic

The pandemic has undoubtedly caused longstanding ill‐effects upon the health of the migrant population, not only in worsening mental (Blukacz et al. 2022) and physical health (Pettersson et al. 2023) but in their engagement with health services. The pre‐existing lack of social protection measures in place for migrant groups, coupled with ill‐considered pandemic policies that furthered exclusionary practices (Petersen and Pienaar 2024), resulted in failure to engage with services or seeking alternative means of support. In some cases, the move to digital services also posed an additional obstacle for migrants due to language barriers (Knights et al. 2021). The lack of easily accessible information for migrants in their language and a lack of trust in host country institutions during the pandemic was found to lead to reliance on friends and social networks, which often caused the spread of misinformation and fear‐mongering (Page et al. 2022; Zlotnick et al. 2022). Additionally, utilisation of social media for information was found to increase negative attitudes towards the pandemic and caused many to be hesitant of the COVID‐19 vaccine (Thornton et al. 2012). Specifically, regarding vaccine uptake, barriers such as fears of immigration checks prevented many undocumented migrants from seeking the COVID‐19 vaccine (Mengesha et al. 2022), as well as the lack of strong relationships between healthcare providers and migrants which led to a lack of reassurance regarding the efficacy of the vaccine and possible side effects (Lin 2022).

In addition, the diverse characteristics of migrant groups, the importance of social interaction, difficulties in social distancing, and concerns regarding vaccines, were ill‐considered in the creation of measures. Communication that specifically targeted migrants with irregular status was often not effective. Migrants in the UK (Deal et al. 2021), for example, had a significant lack of knowledge or confusion in regard to information affecting their immigration status and residency. Finally, some studies show that public vaccine campaigns disregarded the root causes of historical mistrust in institutional healthcare (Smart et al. 2024), whereas grouping migrants under one category (such as ‘non‐White’ in the UK) highlighted the lack of consideration of the heterogeneity of these populations and led to scientifically inaccurate data (Aspinall 2021).

### 2.3 Institutional Trust and Healthcare

It has been emphasised that trust is one of the most significant barriers to medical care for migrants (Thompson et al. 2004; Yazdani et al. 2024) and may be exacerbated by factors such as experiences of institutional violence, suspicion of government motivations, and a desire to stay ‘under the radar’ due to fear of deportation (Mengesha et al. 2022; Mona et al. 2021). Fear of disclosing personal information has been noted to be a consistent obstacle to migrants' engagement with healthcare due to concerns regarding immigration status—an issue which is particularly salient among undocumented migrants (Biswas et al. 2011; Torres‐Cantero et al. 2007). The need for formal documentation to access healthcare is often a deterrent to access (Karl‐Trummer et al. 2010; Rassa et al. 2021) and causes many migrants to self‐medicate, avoid seeking care, or find alternative forms of healing within their own communities (Gilbert et al. 2019). There are also concerns over treatment costs when mainstream employment was not possible due to legal status (Hargreaves et al. 2020) as well as challenges with communications, continuity of care and confidence—the so called ‘3C model’ (Brandenberger et al. 2019).

Regarding confidence, the inability to develop mutual trust with practitioners, and the perceived lack of control over the process of receiving care, has been identified as a major difficulty in healthcare delivery for migrant groups (Piotr 2007; Rosano et al. 2017). Healthcare interactions are often further complicated by language barriers which make trustful relationships with patients harder to create and maintain (Biswas et al. 2011; Kankaanpää et al. 2024) and contribute to poorer health outcomes as some migrants do not complete treatment courses (Banzola et al. 2020). Finally, fear of racism and hostility from healthcare workers is a key issue preventing migrant access to healthcare (Kapadia 2023; Jiménez‐Lasserrotte et al. 2023). This reduced their willingness to seek help and therefore increased negative mental and physical health outcomes during the pandemic. In migrant lives which are often ‘characterised by disrespect’ through experiences of xenophobia and racial discrimination (May et al. 2008), mutual trust and respect within healthcare interactions is vital to ensure long‐lasting productive relationships.

### 2.4 Risk and Inequality: Reflections on Beck's Conception of the Risk Society

As argued by Beck (1999), the post‐modern world composes of ‘cosmopolitan communities of global risk’ where class divisions were replaced by uncertainty and vulnerability to risk (Beck 1992). It is individualisation linked with neoliberal shift, argued Beck, that is the main driver of change, especially in so‐called ‘post‐welfare’ capitalism. In this context, some studies examining COVID‐19 analysed the pandemic as the ‘cosmopolitan moment’ of world risk society, referring to the levelling effects of the virus (e.g., Chan 2023).

Although we utilise Beck's reflections on the relationship between risk and social structure, there is a lack of consideration in his work of the effects of horizontal divisions that become especially salient in regard to the intersecting identities of migrants. Giritlu Nygren et al. (2024) have emphasised the need to extend Beck's theory and consider risk through the lens of intersectional and feminist theory, whereas Engel and Strasser (1998) explored how vertical divisions such as socioeconomic inequalities are not reduced; rather, structural effects are now more dependent on horizontal criteria such as gender, religious denomination or ethnicity which are also often intersecting. Curran (2016), building on Beck's work, has noted that there are growing social, environmental and financial risks worldwide that serve to intensify inequality and the prevalence of class conflict in post‐modern societies. The possibility of such conflicts also goes against Beck's assumption of linear change in the decline of class (Hanlon 2010). Furthermore, in the relation to catastrophic events, it has been argued that class plays an important role in preventing exposure to risk itself (Bertilsson 1990) as well as sheltering from impacts, such as loss of income in the aftermath of such events (Rose 2000).

The world now is experiencing ‘manufactured’ risks that are produced or mitigated by humans and, while COVID‐19 was not manufactured, awareness of its dangers was negotiated through democratic processes involving scientists, the media, political actors and citizens, resulting in negotiated forms of knowledge. In relation to modern risks, Beck (1992) posits that class structures enable or disable access to knowledge and therefore mediate against risk. Migrants, however, especially those without secure immigration status, often exist outside of these structures and therefore are perceived as having limited access to knowledge, thus cannot be trusted. This can be exacerbated by the inherently racist and xenophobic social structures which arguably impact on migrants' relationship to knowledge and mutual trust more than class structures.

In Beck's final work (2016) he posits that in modern times, risks are inherent within a dynamic, global framework that, instead of only having negative effects, allows for global collaboration in the face of hazards for the common good. Within the context of the pandemic, the globalised nature of risk can indeed be seen as enabling collaboration across geographical boundaries. However, as will become evident, the lack of consideration for the most marginalised in society did not cause a shift in global values of equality, but rather highlighted gaps in provision and lack of mutual trust between European societies and its migrants, emphasising the ‘us‐them’ divide. Although some argued that ‘the virus does not discriminate’ (e.g., Ozturk 2021), there is strong evidence that the most prominent exemplification of the risk society, those in precarious employment, were more at risk. In such cases, evidence shows that Beck's thesis of the ‘equalising nature of risk distribution’ proved to be flawed during the global pandemic, with inequalities increasing and migrants particularly vulnerable.

This article will therefore build upon these more recent connections between risk and inequality and emphasise the ways in which migrants' relationship with trusting others and being trusted themselves affects the ways in which they experienced and mediated risks during the COVID‐19 pandemic.

## 3 Methodology

The RESISTIRÉ Project explored the impact of the COVID‐19 pandemic on behavioural, social, and economic inequalities in Europe through data collected by a network of national researchers (NRs) in 30 European countries. The data collection consisted of three consecutive cycles (July 2021–December 2022). The aim in these cycles was to collect diverse experiences highlighting the intersection of inequalities and identities. The project was proposed through a multi‐disciplinary consortium of 11 partners from 9 European countries and brought together policy analysis, quantitative and qualitative research activities to investigate, analyse and monitor the impact of COVID‐19 in different domains and on various inequality grounds. It was underpinned by a gender+ approach that ‘recognises that gender inequality and other inequalities are connected and are thus best addressed with those possible intersections in mind’ (Verloo et al. 2011, 4).

One component of the qualitative research conducted was narrative interviews with members of marginalised groups (for further discussion of the use of narrative interviews in the RESISTIRÉ project, please see Delaney et al. 2024). Thirty national researchers (NRs) were contracted to undertake these interviews and in total, 740 narrative interviews were conducted. The study adopted purposive sampling, and participants were selected from marginalised groups characterised by intersecting inequalities. For each interview, NRs indicated which characteristics were apparent grounds of inequality in the narrative. They could choose from a list based on the protected grounds in EU anti‐discrimination law or select ‘other’ and specify. Multiple selection was possible. According to this estimation, migration status most often intersected with social class (61%), gender (45%) and race/ethnicity (33%). Age was selected for 21% of the narratives and disability, sexuality, gender identity and religion were all less than 10%. Of those interviewed, 108 were migrants, broadly defined here as individuals currently living in a country other than their country of birth. As can be seen in Tables 1 and 2, the sample was largely drawn from female migrants, due to the purposive sampling of the project, and the vast majority were of working age.

**TABLE 1:** Gender distribution of interviewees.

Gender	*N*	Percentage (%)
Woman	81	75.0
Man	25	23.1
Non‐binary	2	1.9

**TABLE 2:** Age distribution of interviewees.

Age groups	*N*	Percentage (%)
19–29	27	25
30–39	30	27.8
40–49	31	28.7
50–59	12	11.1
60–66	5	4.6

The interviewees originated from a variety of geographical locations (Table 3) and resided in a range of European countries (Table 4). Although narrative interviews were conducted in 30 countries, migrants are not represented in all national samples as NRs were given considerable freedom in selecting participants accessible to them and who they deemed vulnerable within their national context.

**TABLE 3:** Geographical distribution of interviewee home countries.

Region of origin	*N*	Percentage (%)
EU	18	16.7
Europe other than EU	12	11.1
Americas	20	18.5
Middle East	13	12.0
Asia other	14	13.0
Africa	19	17.6
Unclear origin	12	11.1

**TABLE 4:** Number of migrant narratives per host country.

Host country	Number per country
Hungary, Lithuania, Poland, Romania, Serbia, Slovakia, UK	0
Finland, Slovenia	1
Austria, Belgium, Bulgaria, Croatia, Cyprus, Czechia, Denmark, Estonia, Germany, Iceland, Luxemburg, Latvia, Netherlands, Portugal, Spain, Turkey	3–6
France, Greece, Ireland, Italy, Sweden	7–10

The narrative interviews were analysed using qualitative data analysis software, combining deductive and inductive approaches. The initial focus was on (1) access to health services, and the data was coded based on hinders identified in previous research. Additional codes emerging from the data relating to health were collected within specific topics, and two of these ‐ ‘risk’ and ‘trust’—led to closer secondary analysis. This resulted in two additional themes: (2) risk through lack of agency and exposure to the virus, and (3) risk of exclusion as the result of mutual distrust. Taken together, these interconnected themes problematise Beck's concept of ‘widespread’ risk in the context of the pandemic and call for a more nuanced understanding of trust as a mutual process.

Informed consent was obtained from all participants, all interviews were fully anonymised, and names used in the analysis are pseudonyms. Ethical approval for this study was obtained from Oxford Brookes University Research Ethics Committee (UREC 201492), TU Dublin (REC‐20‐122) and from the Swedish Ethical Review Authority (2021‐02535).

There are some considerations regarding the limitations of this study as, due to the nature of the project, the narratives are drawn from different countries with diverse migration patterns, social attitudes and healthcare systems. This article does not intend to explore the specificities of national contexts but rather examine the themes and inequalities that cut across these contexts. Participants' experiences also greatly differed as, whereas migrants often share similar characteristics, they are highly heterogeneous groups.

## 4 Findings

The pandemic and related lockdowns resulted in extreme challenges faced by all citizens. In particular, changing rules, restrictions, and access to services (including health), combined with fear of the virus, were unprecedented. Although all suffered from the ongoing crisis, the narratives conducted for the purpose of this project reveal that migration status constituted an additional challenge. Specifically in relation to health, we identified the following three themes that emerged as important in the narratives with migrants. These were (a) access to health services, specifically in relation to financial barriers and a lack of information; (b) lack of agency in relation to the exposure to the virus and (c) issues related to mutual trust between migrants and the host country population.

### 4.1 Access to Health Services

Although access to health services became very restricted for all during the COVID‐19 pandemic due to safety measures, migrant populations experienced additional difficulties, even before the crisis. With limited access to publicly funded healthcare, migrants often had to pay out‐of‐pocket, and, when combined with an economically precarious situation, this lack of *affordable* healthcare was a considerable barrier. Valeria, originally from Russia but living in the Czechia, described feeling like a ‘second class citizen’ because of the difference in access to healthcare long before the pandemic:

As non‐EU migrants with long‐term residency, we are not part of the public healthcare system and have to pay for our private insurance. There have been many instances when it was difficult for us to register our children to specialists […] or when we had to visit an emergency with them, and we were treated with disrespect and suspicion.

Inequalities, however, became even more apparent during COVID‐19 in relation to the vaccine. This is how Valeria describes her family situation:

The vaccination for migrants with long‐term residency regardless of their age or health status opened at the beginning of June 2021—long after the registration was open for all the Czechs over 18 years old [and EU citizens and migrants with permanent residency]. I had to pay upfront, which was I reimbursed by my private insurance […] for migrants who work as general low‐skilled labour, this might be money they cannot spare.

Valeria spoke from a relatively privileged position, and she saw equal access to healthcare as a matter of principle. To several other narrators, the cost of healthcare meant delaying seeking treatment or avoiding it altogether. Entitlements did, of course, also vary depending on the regulation in the host country in question, and the residence status of the narrator.

In addition to a lack of affordability, our data point to difficulties related to information about accessing services during the time of crisis. This was in addition to difficulties navigating the national health systems prior to the pandemic. The following quote from Flore, an American woman living in Italy with her husband Paul, illustrates this issue:

The way the health system works here is very confusing and cumbersome, there is no sort of centralised database or anything. You run from one office to another and many times you get a lot of wrong information from the personnel at the various offices. All‐in‐all, it took a year for the health system to realise what was wrong with Paul. By then, he had stage four throat cancer. […] if you are a foreigner, there is really no one to support you if you are not fluent in Italian, to help you navigate through the labyrinth of the healthcare system.

As the quote demonstrates, language was an additional barrier. Not only was information often conflicting, but language barriers also impacted migrants as the situation in regard to lockdown and restrictions was rapidly changing. Informal networks proved to be of help in such cases. Charlotte, a Filipino woman living in Belgium explained:

While still difficult for someone who speaks English, I am now less anxious about navigating the health system than before, thanks in large part to the health insurance card for newcomers (that I am now aware of), and also due to my partner who can speak Dutch. In general, I was (and still am) greatly helped by other people with foreign backgrounds who live in Belgium, as they shared a lot of strategies to navigate life here […] For instance, I am part of a specific Facebook group where people can share their experiences and ask each other for advice. […] While being here, I have gradually come to realise that the Belgian bureaucracy is enormously convoluted.

As is evident from the quotes above, the inaccessibility of bureaucratic processes to non‐native speakers often served to increase the marginalisation of migrant groups who were already on the periphery of society. Without the use of informal networks or individual safety nets, many migrants would have been left without assistance, as it was not readily offered by the state.

### 4.2 Lack of Agency—Exposure to Risk

Not only was access to healthcare limited to a greater extent for some of the migrants interviewed for the project, but so was access to benefits and the ability to take sick leave after contracting the virus. This was partially due to their legal status and partially driven by the necessity to have continuous income. The following quote from Ahmet, a Syrian man living in Cyprus, illustrates the latter situation as he continued to work:

During the 1st COVID‐19 lockdown, it was difficult. We had to send an SMS and lie if we were caught. But then, during the next lockdowns, the police were not so many so we could just work after hours and get paid. Another setback during COVID‐19 was when one of the crew got sick with COVID‐19! Even if one got sick, we all had to stay home. […] So, we got by, by lying. If we had COVID‐19 but no or limited symptoms, we just went to work because we could not afford losing the money. I know this is bad. But what could we do? What would my family and me eat if we lost the job?

Ahmet's narrative points to the wider responsibilities of migrants, as he financially supported family members in the host country as well as in Syria. It also provides an example of the type of precarious employment often found at this intersection of social class, race and migration status, often resulting in higher exposure to risks brought by the virus. It is important to emphasise that, in most cases, exposure to risk was driven by financial deprivation—a common issue experienced by migrants, especially those working in precarious or low‐paid jobs—and not by a failure to comprehend the danger of the virus. In relation to measures aimed at preventing the spread of COVID‐19, migrant narratives often contained comparison between the host country and the country of origin, and although conflicting information sometimes led to difficulties knowing what information to trust and which rules to follow, it should be noted that comparisons between countries were not restricted to the migrant narrators. Non‐migrants also made frequent comparisons between the COVID‐19 strategies of different countries, rarely in their own country's favour. Overall, there is little in the narratives that suggests that migrants were less likely to take the pandemic seriously. On the contrary, the uncertainty and the often‐limited access to information seemed to lead to more intense fear. Despite this, restricted access to welfare support in some of the host countries led to a lack of choice or ability to reduce exposure.

The relatively high risk of virus exposure experienced by migrants, as well as their vulnerability to such exposure, was a prominent theme in the analysis. This was two‐fold in nature. On one hand, migrants tend to be over‐represented among essential workers (Fasani and Mazza 2020). On the other, they are also often located at the bottom of the host country labour market with extreme precarity and limited choices in relation to teleworking or not working during lockdowns. For example, Sarah, a Syrian refugee living in Bulgaria, explained her situation in the following way:

Because of the nature of my job, I can’t work remotely. I always have to be in the office. So even in the highest peaks of the pandemic, I have been going to the office just because I had to keep my job and the only finances. I was scared that I might get ill—but I had no choice […]. But I kept going to work, taking a lot of care to stay healthy, because I needed money.

As illustrated by the above quote, the fear of losing the income necessary to survive in the host country was higher than the fear of virus contagion. Sarah specifically refers to the ‘lack of choice’, which was a common theme among the narratives. This was amplified by occupational status, with those who were live‐in care workers and home‐care assistants even more constrained. The following quote from Maria, a Nicaraguan woman living in Spain, provides an example of such a situation:

The lady caught the virus, but she was not isolated in her room, nor did she use the mask. I had to get her up, move her and do the household chores. Nobody cared about my health. I tested negative but she took a long time to test negative. I wanted to quit my job, giving them notice, but this woman's family wouldn't let me leave because they said they couldn't find anyone to take care of this person being positive, and that I was obliged to stay there because I was living with someone who tested positive. […] I felt practically kidnapped, and I didn't know if they were right. I would have liked to have a phone number to get information or know someone who could advise me on my rights, but I was afraid of being reported. We come here to work, but as it is not our country … you always feel afraid. We, migrants, live a double life: we take care of the elderly here, but we also have to take care and support our families there.

This quote, in addition to her lack of choice, also touches upon two other important themes: the lack of access to information and the fear of being deported. Accessing information about the rights of non‐citizens, relating to both healthcare and work, was a common concern among migrants interviewed, especially affecting those with limited knowledge of the host country language. The fear of getting deported also constituted a common barrier for those with undocumented status. It should also be noted that the experiences of racialised migrants such as Sarah and Maria are often characterised by greater levels of mutual distrust due to discrimination, institutional racism and histories of colonialism. These issues contributed to the lack of trust from migrants and host countries, which is the final theme identified through analysis.

### 4.3 Mutual Forms of Distrust

In the narratives, different aspects of ‘trust’ were discussed in relation to the pandemic. These can be classified as follows: (1) trusting healthcare systems and professionals, (2) being trusted by healthcare professionals and (3) trust in a wider social context. These three themes will be scrutinised in the analysis presented below.

#### 4.3.1 Trusting Healthcare Systems and Professionals

The narratives contain several examples of migrants delaying medical treatment for financial reasons as it was a cost they could ill afford, however there were also instances of migrants fearing negative consequences of seeking medical help. This was the case of Carla, originally from Honduras. Her irregular status in Spain meant going for cancer treatment was fraught with fear:

I was afraid when I went to the hospital for the treatment, because there was the lockdown and the police was controlling people in the street, but this was a life‐or‐death situation, and I thought that the sense of humanity would prevail on the law.

In the end, humanity did prevail, and Carla did receive treatment. She also managed to secure a job that entitled her to a residence permit. However, this meant working whilst receiving cancer treatment to maintain access to care, which was extremely difficult for her.

Threat of deportation also overshadowed many migrants when seeking healthcare. For Alex, an asylum seeker in Iceland, the fear of deportation caused him to not take the vaccine:

I'm not vaccinated even though I really want to receive the COVID‐19 vaccine, because the authorities are using the vaccination to deport asylum seekers. It has happened before where the authorities weaponise the vaccine to deport and scare other asylum seekers. I can’t let that happen to me because I have no place to go to. I'm scared for my health to not be vaccinated and therefore risk catching the virus […] I don’t want to put other people's lives in danger so I've been isolating myself a lot. This is a matter of basic human right to have access to medical care.

It is important to note that Alex's fears were not unfounded, as Icelandic media reported at the time that vaccination certificates were used by the police to locate and deport asylum seekers (Fontaine 2021). This ‘weaponisation’ of healthcare during the crisis reduced migrant engagement with health services not only during the pandemic, but potentially for a long time to come.

Although fear of deportation did not affect all migrants, a general sense of mutual distrust was a prevailing theme in the narratives, as migrants themselves described not feeling trusted within wider society. These feelings of mistrust are reflective of studies that have explored migrant and ethnic minorities' experiences of healthcare outside of the pandemic, finding that many feel marginalised, misunderstood and ‘othered’ by healthcare professionals (Subramani 2024; Bradby et al. 2020).

#### 4.3.2 Being Trusted by Healthcare Professionals

Trust is a mutual process, and while several migrant narrators described a difficulty trusting professionals, there were also those who described not being trusted in a healthcare setting. Valeria, cited at the start of this section, described often being treated with ‘disrespect and suspicion’ when seeking medical care. In her case, exclusion from the public healthcare system undoubtedly added to poor treatment, as migrants were not seen as rights‐holders but as individuals whose ability to pay for the treatment had to be assessed. There are also stories where the sense of distrust and lack of respect was expressed in terms of not being taken seriously when voicing health concerns. Viktoria, a migrant women living in Estonia, described her difficulties accessing healthcare as follows:

My daughter actually developed a panic disorder over the COVID‐19 period, so she was not able to attend school even when it would have been possible in the autumn of 2020. […] We started with consulting our general practitioner, but she was rather helpless, did not really have any suggestions for what to do or who to see. Interestingly, when my husband went there with our daughter, it was easier to get the appointments and referrals.

Viktoria is married to an Estonian man, and her story shows how healthcare experience not only differed due to nationality and ethnicity but was also gendered. In this case, the intersecting inequalities could be linked with trust, as health services were more likely to engage with a man with no‐migration background. To an extent, this could also be related to the place occupied by migrants in the space of ‘reliable knowledge’, in relation to medical science and being ‘trusted’. In this case the ‘experts’ (healthcare professionals) did not perceive her as trustworthy of understanding specialist knowledge as she represented the ‘other’ who is located outside of that reliable knowledge space. A similar experience was shared by Florence, a Kenyan woman married to an Irish man. The quote illustrates this point:

I was not given any information about why they did a caesarean; I don’t think that would have happened to an Irish woman. When I insisted that my husband was sitting in the car outside, he was called in but that was after the caesarean. They apologised to him but not to me. The apology was given to the man not the woman who had the baby.

Once again, the experience of not being trusted with information was intersectional with both gender and race as factors. This narrative, although also pointing to a wider issue of restrictions in maternity care during the pandemic, emphasises how intersecting inequalities of gender and migration can combine to reduce level of care provided in healthcare settings.

#### 4.3.3 Mutual Trust in a Wider Social Context

It is important not to view healthcare‐related trust in isolation, as the issues identified within healthcare systems are reflective of a wider social context where lack of mutual trust is prevalent. These tendencies were put in a sharper focus during the pandemic. Many migrants described living in societies that treated them as disposable workers and viewed them with suspicion as ‘virus spreaders’. Oleh, a Ukrainian man living in Estonia described how, before the outbreak of the war, migrant workers from Ukraine were blamed for spreading the virus:

The stranger is always the easiest to blame. The locals did not care what we felt, how we got tests or how we supported ourselves. They had no idea how hard life was back home and how hard we worked.

Two interconnected themes found in Oleh's narrative occur in several other narratives. One is the perception that ‘they’ do not *trust* ‘us’, and the other is that ‘they’ do not *care* what happens to ‘us’. These sentiments are perhaps most poignant when expressed by the several migrant care workers interviewed. Maria, who was cited above stating that ‘nobody cared about my health’, added that ‘people are more distrustful or think we are not responsible persons, because we are migrants’. Karina, a care worker from Armenia living in Turkey, echoed this sentiment:

Here, first of all, they think that the worker will infect them. The neighbour of my employers was infected, and assumed that they were infected from their worker, but in the end, it turned out that a family member was sick.

A final quote from Christina, a migrant care worker from Albania living in Greece, illustrates how this interconnected lack of mutual trust and care is expressed in a care work setting:

I was very careful and always took care to sanitise everything, wear a mask, make sure that I am not in the same room as my employers. However, my greatest problem is that my employers are not as careful. They expect me to wear a mask and keep everything sanitised, but they do not wear a mask. […] my work makes it very difficult to be careful because the clients ignore us. They do not care about our health. They only protect themselves.

Although these narratives emphasise the lack of trust placed in migrant workers during the pandemic, they also show a lack of respect and care for them. The expectation to protect others was often seen as one‐sided rather than a mutual obligation, reflecting wider societal attitudes towards migrant populations.

## 5 Discussion

This paper has explored several themes regarding migrant experience in Europe during the COVID‐19 pandemic. It contributes to existing literature on the relationship between migration and healthcare both during the pandemic and more broadly, as the crisis merely exposed the pre‐existing challenges and barriers experienced by migrants in their access to, and experience of, forms of healthcare. These findings have added narrative depth to the fact that more migrants were at risk of exposure due to their occupational status and has explored their entitlement to healthcare and welfare which is often tied to their residential and employment status (Miles et al. 2024). Such ties therefore meant employment was paramount in migrant lives as the basis upon which other aspects of their existence was dependant, and the question of whether going to work was ‘too risky’ due to the chance of virus exposure, became replaced with ‘can I afford not to work?’. It is also evident that although migration status alone is often a barrier to information and affordable healthcare, when migrant identities intersect with issues of class and ethnicity/race these barriers can become even more unsurmountable and can increase both exposure to risk and lack of mutual trust. Although the literature acknowledges that the pandemic brought to light the pre‐existing inequalities for many marginalised groups (Sandström et al. 2022; Harroche et al. 2023), it is important to explore the ways in which migrants' experiences were also influenced by their social interactions with host country citizens, and by the way they were perceived by members of the host country population through the lens of trust.

During the pandemic, there existed an omnipresence of risk for all citizens, which highlighted the ways in which individuals had to navigate their world while measuring these risks against potential dangers and benefits (Beck 1992). Nevertheless, inequalities in relation to risk exposure are also structured (Beck 2007). Our study contributes to the problematisation of risk theory in the consideration of migrant experience, which is often affected by multiple, intersecting identities and, subsequently, multiple intersecting risks. Beck's (1992) notion of the risk society as ever‐shifting in light of new hazards is evidenced clearly within the COVID‐19 crisis. It has revealed fallacies and misconceptions within existing social, economic and health structures which cause the most marginalised, including many migrants, to experience greater risk. Many countries not only failed in their provision of COVID‐19 regulations advice in multiple languages, but also created bureaucratic and digital barriers, and offered ill‐suited guidance to migrant groups (Aspinall 2021; Smart et al. 2024). Institutional attitudes towards migrants in core messaging and engagement deepened concerns over racism and xenophobia that had long existed in healthcare provision, which served to increase feelings of mistrust and often prevent migrants from accessing healthcare when needed (Smart and Weiner 2018). The lack of consideration of migrant needs in the provision of healthcare or other support therefore left many feeling isolated and further marginalised in a liminal space where they were not valued for their contributions and were disparaged for their lack of ability to follow pandemic regulations. Within this study, there was very little to suggest that narrators were uninformed about the dangers of the virus, despite prolific messaging of migrant groups as ‘virus spreaders’. However, it is evident that several took risks despite knowing the dangers for fear of losing their residence or employment, which follows the evidence from international literature (e.g., Orton et al. 2022). Migrants were therefore often caught in a double‐bind, with their inability to engage with pandemic regulations due to language difficulties, bureaucratic barriers, residential status discrimination and financial situations meaning they were ill‐trusted. In this context, migrants were regarded as (1) constituting a risk as unreliable ‘virus‐spreaders’ and (2) those who were much needed, but not valued, workers expected to take greater health‐related risks, often in front‐facing and key worker roles. The relationship between risk and trust has become complex as the unequal distribution of risk impacted migrants who were ‘not trusted’; yet, they themselves did not trust that they were safe in the host society in relation to contracting the virus or safe access to services.

In this context, risk can be further linked to ‘trust’ which is a key concept in this article. Risk awareness is related to knowledge and migrants are typically framed through a ‘migrant deficit model’ where migrants are at risk because they are less likely to trust authorities and are potentially less able to distinguish information from disinformation (Roura et al. 2021). Our study has, however, shown that the health implications of *not being trusted* as knowledge holders can be equally, if not more, severe. In our contribution to the existing literature on the sociology of health and illness we thus demonstrate how this level of mutual distrust served to widen gaps in healthcare provision and may have long‐lasting effects on the ability and willingness of migrants to engage with medical services in the future. These long‐term implications would thus require more research scrutiny in the post‐pandemic world.

This paper has therefore examined the interplay between trust, inequality and health in times of crisis, and emphasised how the narrative of COVID‐19 as a levelling experience within societies, fails to recognise the intense barriers for migrants associated with immigration laws, bureaucracy, language and discrimination. Our analysis has moved away from the paradigm of ‘methodological nationalism’ (Beck 2007) with pan‐European perspectives adapted instead; thus, showing the future need for focused examination of migrants' societal position across European societies within further research.

## Author Contributions

**Alexis Hawthorne:** conceptualisation (equal), formal analysis (equal), methodology (equal), writing – original draft (equal), writing – review and editing (equal). **Alicja Bobek:** conceptualisation (equal), formal analysis (equal), methodology (equal), writing – original draft (equal), writing – review and editing (equal). **Lina Sandström:** conceptualisation (equal), formal analysis (equal), methodology (equal), writing – original draft (equal), writing – review and editing (equal).

## Ethics Statement

Ethical approval for this study was obtained from Oxford Brookes University Research Ethics Committee (UREC 201492), TU Dublin (REC‐20‐122) and from the Swedish Ethical Review Authority (2021‐02535).

## Conflicts of Interest

The authors declare no conflicts of interest.

## Data Availability

The data that support the findings of this study are available from the corresponding author upon reasonable request.
